
JOURNAL INFORMATION
==============================
NLM Title Abbreviation: ScientificWorldJournal
Journal ID: ScientificWorldJournal
NLM Journal ID: 101131163
Journal ID: TSWJ

The Scientific World Journal

ISSN: 2356-6140
EISSN: 1537-744X
Publisher: Wiley

ARTICLE INFORMATION
==============================
PMCID: PMC5823108
PMID: 19468657
DOI: 10.1100/tsw.2009.56
Article ID: 254145
Article version: 1
Subjects: Commentary

About “Low Mortality and Key Aspects of Delivery of Care for End-Stage Renal Disease in Italy”

Pascual Julio 1 *
Portolé Jose M. 2
Lloveras Josep 3
1 Servicio de Nefrología, Hospital Ramón y Cajal, Universidad Europea de Madrid, Spain
2 Servicio de Nefrología, Fundación Hospital Alcorcón, Madrid, Spain
3 Servei de Nefrologia, Hospital del Mar, Institut Municipal d'Asistencia Sanitaria, Barcelona, Spain
*Julio Pascual: julpascual@gmail.com
Publication date: 2009
Electronic publication date: 2009 May 22
Volume: 9
First page: 360
Last page: 362
Received 2009 May 20; Revised 2009 May 21; Accepted 2009 May 21
Copyright: Copyright © 2009 Julio Pascual et al.
Copyright year: 2009
License: This is an open access article distributed under the Creative Commons Attribution License, which permits unrestricted use, distribution, and reproduction in any medium, provided the original work is properly cited.
License URL: https://creativecommons.org/licenses/by/3.0/

==============================
Commentary
*TheScientificWorldJOURNAL* (2009) 9, 360–362
ISSN 1537-744X; DOI 10.1100/tsw.2009.56

TheScientificWorldJOURNAL
www.thescientificworld.com

### About "Low Mortality and Key Aspects of Delivery of Care for End-Stage Renal Disease in Italy"

Julio Pascual[1,*], Jose M. Portolés[2], and Josep Lloveras[3]

*[1]Servicio de Nefrología, Hospital Ramón y Cajal, Universidad Europea de Madrid, Spain; [2]Servicio de Nefrología, Fundación Hospital Alcorcón, Madrid. Spain; [3]Servei de Nefrologia, Hospital del Mar, Institut Municipal d'Asistencia Sanitaria, Barcelona, Spain*

**E-mail**: julpascual@gmail.com; jmportoles@fhalcorcon.es; JLloveras@imas.imim.es

*Received May 20, 2009; Revised May 21, 2009; Accepted May 21, 2009; Published May 22, 2009*

---

The article by Lauder et al.[1], just published in *TheScientificWorldJOURNAL,* provides an analysis of the results of treatment for end-stage kidney disease in Italy and explores the possible reasons for the excellent outcomes. It is based on data collected in a survey of about 25% of the Italian dialysis centers with an annual mortality of 12%, which is far below the European mortality rate and strikingly lower than the mortality rate in the U.S.

This report has some methodological limitations, such as lack of analysis of the association between factors proposed as causative (vascular access, predialysis follow-up, etc.) and the observed effect (low mortality). It also does not take into account the error of positive selection, such that centers with the best results may have been more likely to respond to the survey. The fact that the mortality rate is slightly lower than the one reported by the Italian registry suggests that this may have been the case. Despite all of this, this report establishes a reference of what one could achieve in terms of optimizing results among patients on hemodialysis as a chronic replacement therapy.

The comparison of results among national registries or between countries participating in multinational studies (such as DOPPS[2]) allows identification of better outcomes and exploration of the reasons. At stake is the task of identifying the elements, often organizational in nature, in terms of health care delivery, which promotes admirable results, which is precisely the aspect that is highlighted in the article by Lauder et al.

The authors of this article acknowledge that one cannot fully control for different characteristics of the population studied, such as ethnicity[3], prevalence of diabetes mellitus, obesity, and differences in life style. In fact, the life expectancy in the population at large is higher in Mediterranean countries than in northern European countries, and this could also extend to the dialysis population. The results in Spain, another Mediterranean country like Italy, are also better than the European median and from North America, but not as good as those here reported for Italy. The Spanish environment is similar, with an incidence of diabetes mellitus of about 20%, a similar range of age, and a public health care system where access to care is available universally.

In a prospective study of 1-year duration including 1,700 patients on hemodialysis (prevalent patients), the mortality rate was only 4%[4], and in another study recently completed, including 2,100 patients (incident patients), the mortality rate was 11.8% for the first year and 19% after 2 years, all below the 13.5% annual mortality based on the Spanish registry[5].

The authors propose that certain elements of the Italian health delivery system are responsible for the excellent results observed in this country. It is not the first time that the organization of the health delivery

*Corresponding author.
©2009 with author.
Published by TheScientificWorld; www.thescientificworld.com system and the focus of medical care played a role beyond other metrics of quality, such as the achievement of the goal hemoglobin level or the kT/V. In fact, as noted in the paper by Lauder et al., patients from the U.S. have better indicators of provision of effective dialysis and anemia management, but yet the mortality rate is higher than in Italy. The authors of this landmark survey center their analysis in aspects related to the delivery system of nephrological care in Italy, which is largely a public one. In this model, it is noteworthy that there is an integral approach to care, starting at the early phases of kidney disease. Seventy-five percent of patients are followed from phases CKD 3 and 4, and the models of provision of vascular access are integrated as well (about 25% involve an integrated approach between nephrologists and surgeons).

The follow-up of CKD from its earliest stages allows for adequate preparation of the patient prior to renal replacement therapies and is an element that has already shown to lead to good results[6]. The survey of the Italian system provides results that are above any other reference of which we are aware. In 1999, it was estimated that about half of patients in dialysis in Spain had been previously evaluated in consultation prior to dialysis[4]. In fact, it is noteworthy that the glomerular filtration rate at the initiation of dialysis was at about 10 ml/min. Although we do not have this information available at the majority of registries, one could think that initiation of dialysis early on contributes to low mortality rate. In CKD stages 3 and 4, one can also identify differences among countries[7]. In a Spanish study based on 1,100 patients followed for 5 years by nephrologists, the percentage of patients entering replacement therapy was 19.2% at 3 years and the mortality rate was 10.4%. It seems that the differences are already present at the predialysis phase regarding both population differences and during an integrated follow-up[8].

In the article by Lauder et al.[1], there was very little attention paid to home dialysis, fundamentally peritoneal dialysis, such that mortality and other details are not mentioned. In general, those patients are about 10 years younger and have less comorbidities than those on hemodialysis[9,10]. In a collaborative Spanish study that involved over 450 patients on peritoneal dialysis, mortality was 5.4%, a figure below the Spanish registry[8,9], reflecting a selected sample from centers committed to this type of dialysis (an example of positive selection). The number one cause of termination of this mode of dialectic therapy is kidney transplantation, which occurs in 24% of patients that have been on peritoneal dialysis for a year.

The public health systems in the Mediterranean countries have a relatively high number of nephrologists per hundred thousand people. The task of the nephrologists in this environment revolves around teamwork and the frequent presence in dialysis treatment rooms. This accessibility may provide results that go beyond the careful clinical follow-up and the accomplishment of the “cold” goals and objectives that are part of the dialysis quality of care plans. Some authors have noted the importance of the amount of time spent in the dialysis center and with the patient; the infrequent number of visits by the dialysis physician was associated with a significant increase in mortality[11]. This conviction takes us back to the basics in the practice of Medicine: that the close relationship between the physician and the patient, at a time of technological dominance, continues to be fundamental to achieve good therapeutic results.

### REFERENCES

1. Lauder, A., Schieppati, A., Conte, F., Remuzzi, G., and Batlle, D. (2009) Low mortality and key aspects of delivery of care for end-stage renal disease in italy. *TheScientificWorldJOURNAL* **9,** 349–359.
2. Página: 361
Lopes, A.A., Brag-Gresham, J.L., Ramirez, S.P., et al. (2009) Prescription of antihypertensive agents to haemodialysis patients: time trends and associations with patient characteristics, country and survival in the DOPPS. *Nephrol. Dial. Transplant.* [Epub ahead of print]
3. Peralta, C.A., Shlipack, M.G., Dongjie, F., et al. (2006) Risks for end-stage renal disease, cardiovascular events, and death in Hispanic versus non-Hispanic white adults with chronic kidney disease. *J. Am. Soc. Nephrol.* **17,** 2892–2899.
4. Portoles, J., Lopez-Gomez, J.M., and Aljama, P. (2007) A prospective multicentre study of the role of anaemia as a risk factor in haemodialysis patients: the MAR Study. *Nephrol. Dial. Transplant.* **22,** 500–507.
5. Martín-Malo, A., García, F., Cuevas, X., et al. (2007) Factors associated with mortality in the second year after initiating HD. Results from a multicentre prospective observational cohort study-ANSWER. ASN

6. Gorriz, J.L., Sancho, A., Pallardo, L.M., et al. (2002) Longer pre-dialysis nephrological care is associated with improved long-term survival of dialysis patients. More facts. *Nephrol. Dial. Transplant.***17,** 1354–1355.
7. Página: 362
Keith, D.S., Nichols, G.A., Guillon, C.M., Brown, J.B., and Smith, D.H. (2004) Longitudinal follow-up and outcomes among a population with chronic kidney disease in a large managed care organization. *Arch. Intern. Med*. **164,** 659–663.
8. Navarro, J., Cases, A., Portolés, J., et al. (2009) High hospitalization and mortality rate in diabetic and non-diabetic chronic kidney disease patients (MERENA study). Results of a three year follow-up. *Nephrol. Dial. Transplant.* (EDTA Congress'09)
9. Arrieta, J.A., Gutierrez, G., Moreno, I., et al. (2008) Informe d esituación de diálisis y trasplante en España, 2005. *Nefrología* **28,** 151–158.
10. Portolés, J., Del Peso, G., Fernández-Reyes, M.J., et al. (2009) Previous comorbidity and lack of patient free choice of technique predict early mortality in peritoneal dialysis. *Perit. Dial. Int*. **29,** 150–157.
11. McClellan, W.M., Soucie, J.M., and Flanders, W.D. (1998) Mortality in endstage renal disease is associated with facility-to-facility differences in adequacy of hemodialysis. *J. Am. Soc. Nephrol.* **9,** 1940–1947.

**This article should be cited as follows:**

Pascual, J., Portolés, J.M., and Lloveras, J. (2009) About "Low mortality and key aspects of delivery of care for end-stage renal disease in Italy". *TheScientificWorldJOURNAL* **9**, 360–362. DOI 10.1100/tsw.2009.56.
